# The Welsh Institute of Performance Science: A Decade of Integrated Knowledge Translation in Elite Sport

**DOI:** 10.1007/s40279-025-02210-9

**Published:** 2025-04-29

**Authors:** Camilla J. Knight, Kurtis Pankow, David A. Shearer, Joy D. Bringer, Brian R. Davies, Owen C. Lewis, Stephen R. Woodfine, Liam P. Kilduff

**Affiliations:** 1 Department of Sport and Exercise Science, Swansea University, Swansea, UK; 2 Welsh Institute of Performance Science, Cardiff, Wales, UK; 3 Faculty of Life Sciences and Education, University of South Wales, Treforest, Wales, UK; 4 South Wales Doctoral Programme in Clinical Psychology, School of Psychology, Cardiff University, Cardiff, UK; 5 Sport Wales, Cardiff, Wales, UK

## Abstract

Sport science researchers and practitioners have noted a gap between research and practice for some time. Although several solutions have been proposed, few recommendations have been made for researchers to follow that are based on successful, long-term sport science research programmes aimed at translating research into practice. Therefore, the purpose of this article is to outline a decade of work completed by the Welsh Institute of Performance Science as an institute for sport-based integrated knowledge translation. The aim is to provide a framework for other researchers and institutes to follow when engaging with organisations for long-term research-to-practice partnerships. In addition to outlining the development and functioning of the Welsh Institute of Performance Science to guide others, limitations of the approach used are also presented and considered to facilitate the future development of sport-specific models of knowledge translation, ensuring that the excellent research conducted across the disciplines of sport science effectively impacts the work of practitioners.

## Plain Language Summary

In recent years, there has been an increasing focus on addressing the gap between research and practice within sport science and sport medicine. To date, however, there are few published examples detailing exactly how academic research might be integrated within sports organisations. This article explains how the Welsh Institute of Performance Science has attempted to do this using an adapted knowledge translation approach. Specifically, based on a decade of work, it is apparent that Welsh Institute of Performance Science has helped athletes, coaches, sport organisations and researchers engage in a collaborative process that gets research into the hands of practitioners and makes a substantial contribution to practice while still benefiting researchers.

## Introduction

Sport science is inherently an applied field, with a focus on generating research evidence to understand and enhance sport participation and performance. However, across all levels of sport, researchers have frequently noted a research-to-practice gap, in which research evidence is not being integrated into routine practice [1–4]. There are numerous proposed reasons for the gap, including a perceived lack of emphasis on practical application or generating performance solutions within research studies [2], practitioners having limited access to research articles and time to consume new information [3], a perceived lack of ability of practitioners to interpret and apply research [3], a preference among sport practitioners to use non-academic sources of evidence [5], and a disconnect between the knowledge transfer preferences of practitioners and researchers [6].

Recognising the need to facilitate greater use of research evidence in practice, researchers have increasingly paid attention to knowledge translation (KT) to help bridge the research-to-practice gap in sport [1, 7, 8]. Originating within the disciplines of nursing and health promotion, KT has been defined as, “a dynamic and iterative process that includes synthesis, dissemination, exchange and ethically-sound application of knowledge to improve the health of [populations], provide more effective health services, and products and strengthen the health care system” [9; para. 4]. As is apparent in this definition, KT extends far beyond simply making research papers publicly and freely available or sharing research outcomes in various forms (e.g. podcasts, infographics, presentations) on completion of a project. These elements may be included within KT, but to conduct KT, researchers must explicitly target and tailor knowledge to meet the practical needs of those in the field. In short, KT is a deliberate act to ethically address practical needs and challenges with the best available evidence.

**Fig. 1:**
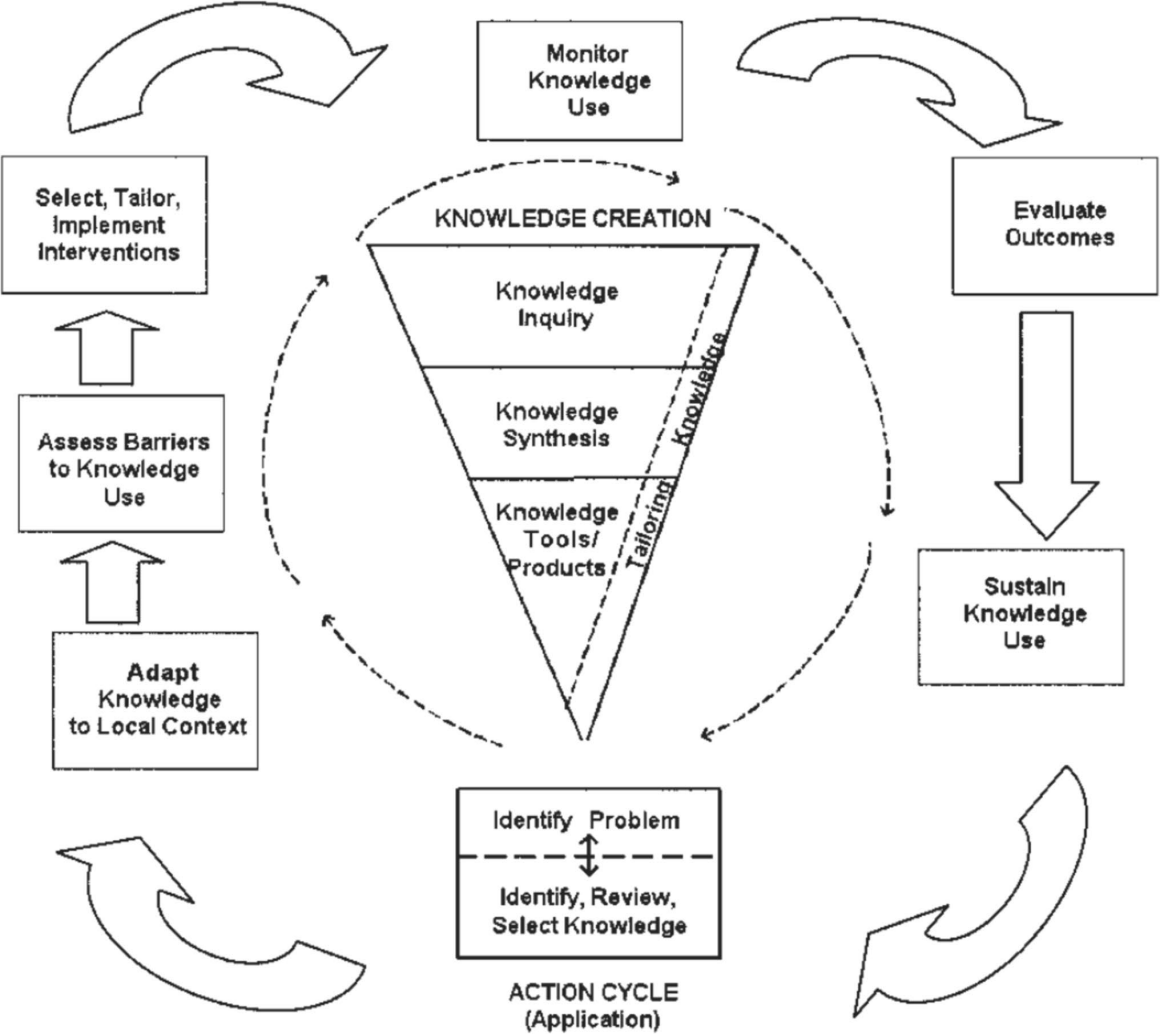
Knowledge to action process. Reproduced from Graham et al. [12] with permission

There are a variety of different models and frameworks available to explain KT processes [10]. Knowledge translation frameworks can be targeted at individual, organisational and policy levels of use, and can be thought of as having one of three aims; providing guidance for the process of KT (i.e. process models); explaining what influences successful or unsuccessful KT (i.e. classic theories or determinant frameworks); and evaluating the outcomes of KT (i.e. evaluation frameworks) [10, 11]. Most common are KT process models that can be used at individual, organisational and policy levels [10]. As an example, a widely used process model of KT is the Knowledge to Action (KtA) framework [12] (Fig. 1). Developed by researchers through a narrative review, the KtA framework is separated into a knowledge funnel and an action cycle. The knowledge funnel is the process of developing primary research (e.g. original studies), secondary research (e.g. reviews) and knowledge products (e.g. programmes, interventions, policies). Knowledge is progressively tailored towards practical application as it progresses through the tunnel. The products from the knowledge funnel then enter the action cycle in which a problem is identified, knowledge users’ barriers to knowledge use are assessed, and knowledge products are tailored, implemented, monitored, evaluated and sustained. The split between the knowledge funnel and action cycle is somewhat artificial, and the authors recognised that the process would likely be iterative and new knowledge could be introduced at each stage.

Most publications regarding KT projects in sport have been conducted by researchers who are located externally to the organisations with whom they are working [7, 8]. To enhance KT efforts and promote evidence-based practices, integrated KT (iKT) is often suggested [13–15]. Integrated KT is thought to improve uptake by addressing several of the potential barriers to knowledge use among organisations, such as a lack of time to interpret research and a lack of understanding of jargon or nuance. Integrated KT has gained attention in sport recently given its potential to provide guidance in co-producing applied research [13, 16]. Specifically, iKT is “an approach to doing research that applies the principles of KT to the entire research process. The central premise of iKT is that involving knowledge users as equal partners alongside researchers will lead to research that is more relevant to, and more likely to be useful to, the knowledge users” [9]; para. 14]. Though many of the issues iKT frameworks address can apply to sport, current models have typically developed for public health and healthcare systems [10]. Consequently, there is limited guidance to support researchers interested in developing full iKT programmes in elite sport settings. Therefore, the purpose of this paper is to introduce, outline and reflect on the work of the Welsh Institute of Performance Science (WIPS) as a process model for iKT in sport to inform practice and future research.

## Welsh Institute of Performance Science (WIPS)

WIPS is an iKT centre that is a joint endeavour between university academics, Sport Wales (the national organisation responsible for developing and promoting sport and physical activity in Wales) and other industry partners (e.g. businesses and other national sporting organisations such as Football Association Wales; see Table 1) [16, 17].

**Table 1:** Organisations involved with the Welsh Institute of Performance Science through projects and/or sharing of best practice and insights

Organisation type	Name of organisation
Political organisation	Welsh Government
National governing body	Sport Wales (Sport Wales Institute), primary link Disability Sport Wales English Institute of Sport/UK Institute of Sport Sport Scotland Institute of Sport Sport Northern Ireland Sports Institute
National sports organisations (not exhaustive list)	Badminton Wales Football Association Wales Hockey Wales Squash Wales Swim Wales Table Tennis Association of Wales Tennis Wales Wales Netball Welsh Athletics Welsh Boxing Welsh Cycling Welsh Judo Association Welsh Gymnastics Welsh Rowing Welsh Rugby Union Welsh Shooting Welsh Triathlon
Universities	Bangor University Cardiff University Cardiff Metropolitan University Swansea University University of South Wales
Other organisations (e.g. charities)	Child Protection in Sport Unit UK Coaching Youth Sport Trust

WIPS was developed in 2014 as a previous knowledge transfer project, the Welsh Elite Performance Sport Innovation Network (WEPSIN), was ending. WEPSIN was a Welsh Government-funded project that sought to generate a knowledge transfer network between Welsh businesses, sport scientists and academics, and high-performance sports organisations. For instance, recognising that heat maintenance (i.e. the maintenance of body temperature for performance during competition where athletes may be inactive and unable to repeat a warm-up) could be an issue for athletes, WEPSIN approached Blizzard Protection Systems, a Welsh-based business specialising in passive heat maintenance, to enquire into their interest in producing heat-maintenance clothing for athletes. Subsequently, sport scientists associated with WEPSIN tested the efficacy of the products produced by Blizzard. These products have subsequently featured at numerous Olympic and Commonwealth Games [18–20].

Recognising the success of WEPSIN and keen to expand upon it, staff at Sport Wales and WEPSIN academics engaged in conversations to discuss an enhanced variation of the project, specifically at the request of Sport Wales, a project that further harnessed the research insights of academics across Wales. Subsequently, WIPS was proposed by the WEPSIN academics to Welsh Government and Sport Wales to “further [develop] sport science in Wales, train future sport scientists, enhance the application of science in Welsh sports and increase collaboration between Welsh sport, academia and business” [17]. Following numerous meetings, presentations and partner discussions, Sport Wales began to provide financial support for WIPS in 2015, as a three-way partnership between Sport Wales, leading academic sport scientists based in Wales, and relevant industry partners.

The funding for WIPS is provided to the two Universities (Swansea University and University of South Wales) to hire research assistants to conduct research and support the work of the Sport Wales Institute, the arm of Sport Wales focused on performance (in contrast to the majority focus on grassroots/community sport within the Sport Wales remit). The Sport Wales Institute is “a team of professionals, from across lots of disciplines, working together to help Welsh athletes achieve success on the world stage—as well as in their lives outside sport” [21, para. 1]. The Sport Wales Institute comprises practitioners from across sport science and medicine, as well as performance advisors who work with national sports organisations (of Commonwealth and Olympic Sports) to assist with their work developing elite athletes. The Sport Wales Institute team also supports British athletes on UK Sport World Class Programmes based in Wales, or when they return to Wales, to deliver at Olympic and Paralympic Games. The WIPS research assistants are university employees but are an integral part of the Sport Wales Institute and have access to Sport Wales offices and systems. There is no financial compensation provided to any other academics engaged in WIPS and there are no other employees of WIPS. Consequently, WIPS functions as an independent research body which can support, upskill, and where needed, challenge the Sport Wales Institute team.

Prior to the establishment of WIPS, the Sport Wales Institute had funded or supported one-off research projects or PhD projects to explore specific research questions. Some previous projects conducted with academics had encountered issues including, (a) academics shifting focus away from the original proposal to serve their own research interests, (b) overly theoretical rather than applied outputs, and (c) projects taking too long and not providing performance solutions in time for major competitions. Consequently, outputs from these projects had not been as beneficial to the Sport Wales Institute team as anticipated and some in the team were hesitant to work with academics who may not understand the pressures that sport organisations are under to deliver on the international stage. Additionally, the ad-hoc funding for individual projects lacked a strategic focus and consequently projects were not always targeted at the most pertinent or pressing performance issues. Sport Wales does have an in-house research and insights team, but their focus is on collecting and managing data on sporting activity in Wales, rather than maximising the performance of athletes.

WIPS sought to enhance the capacity of the Sport Wales Institute by embedding research assistants within the Sport Wales Institute and engaging academics from across Wales to conduct multi-disciplinary, world-leading applied performance science projects that directly addressed the needs of the Sport Wales Institute. WIPS was designed with the intent of producing insights that could be implemented with athletes and wider support teams for immediate performance impact. In developing WIPS, meetings were held with Sport Wales Institute staff, as well as performance directors and coaches from various sport organisations to identify their preferred approach for WIPS to identify, prioritise, and address research questions and performance problems. Through these discussions, five approaches were identified:

(1) Performance-driven questions, science-driven answers. In this situation, athletes, coaches, practitioners or performance advisors will raise questions that are brought to WIPS and subsequently answered based on the existing literature or new research is conducted. This was deemed to be the most common and important role of WIPS for practitioners and performance advisors.

(2) Performance-driven questions, industry-driven answers. As with the above, questions are raised from within the Sport Wales Institute, but answers are sought from business or industry (for instance, data management solutions).

(3) Performance-driven questions, science- and industry-driven answers. When neither (1) nor (2) can provide a sufficient answer, WIPS will seek to combine insights from academics and industry partners.

(4) Science-driven performance applications to enhance performance. This refers to situations in which academics (from within or beyond WIPS) approach Sport Wales/the Sport Wales Institute to gain insights from athletes, coaches, practitioners or performance advisors on performance-focused questions. These questions are only considered if there is a clear benefit to the Sport Wales Institute, and this outweighs any costs to those involved.

(5) Industry-driven performance applications to enhance performance. Finally, in some situations, businesses directly approach the Sport Wales Institute to offer their services and/or test new equipment/technology on athletes/coaches/practitioners. As with academic-driven questions, business/industry needs are only addressed if there is a clear benefit to the Sport Wales Institute for engaging with them.

Priority is given to the first three approaches to research, ensuring that projects that are priorities for Sport Wales (and their athletes and associated national governing bodies) receive the most attention.

**Fig. 2:**
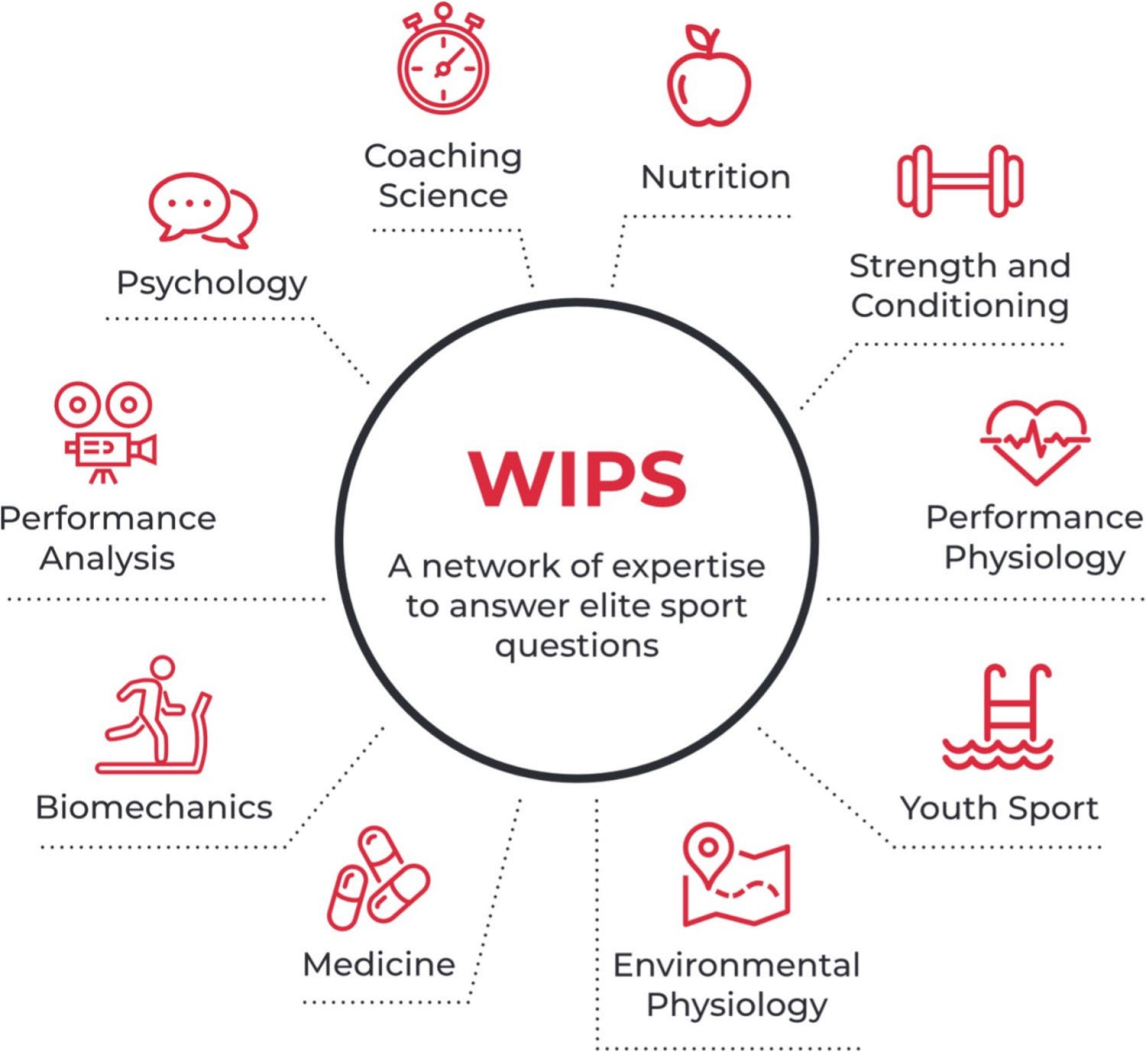
Original performance domains addressed by the Welsh Institute of Performance Science (WIPS) team. Reproduced from [22], with permission

Through these initial conversations, performance areas that were perceived to benefit from specific academic insights and support were also identified. Initially, nine disciplines (biomechanics, coaching science, environmental physiology, nutrition, medicine, performance analysis, performance physiology, psychology, strength and conditioning, and youth sport) were prioritised (Fig. 2), with one academic based at a Welsh University recruited (through open call) to be the academic lead for each area. These disciplines have subsequently expanded to also include data science, disability sport, athlete health and well-being, sports physiotherapy, sports ethics, governance and integrity, and talent identification and transfer.

Structurally, WIPS involves a strategic management board comprising pertinent individuals from Sport Wales, Welsh Government and the academic lead for WIPS. The strategic management board is responsible for providing the overall strategic steer for WIPS and ensuring it remains aligned with its overall purpose. The strategic management board meets annually to review WIPS activities and provide oversight on the Research Steering Group (RSG). The RSG comprises a Sport Wales WIPS lead who shares insights, updates and priorities from the Sport Wales Institute, the WIPS academic lead, WIPS research assistants and academic discipline leads. As noted previously, the academics are not paid for their involvement in WIPS and thus remain independent of Sport Wales/Sport Wales Institute. The benefit (and unique consequence) of this is the Sport Wales Institute has long-term and immediate access to an extensive network of academics who are in full-time academic roles. Therefore the academics have (a) the flexibility to engage in various research projects, (b) access to the latest literature, technology and laboratories required to conduct research, (c) opportunities to apply for and secure funding to support WIPS projects and (d) collaborators who can provide further insights to address performance issues.

As priorities of the Sport Wales Institute have shifted over time, the RSG composition has changed, both in terms of academics involved as well as disciplines included. However, many of the core academics and areas have remained. Of note, the RSG has expanded to include a representative from each of the UK home nations’ sport science institutes (i.e. England, Northern Ireland and Scotland) to share emergent practice and pertinent insights from projects, as well as to facilitate cross-nation working and minimise duplication. Additionally, the RSG has also expanded to include a representative from two national sports organisations outside Sport Wales' oversight (Welsh Rugby Union and Football Association Wales). The RSG meets quarterly to discuss WIPS projects and strategic priorities for each partner group moving forward, and to share pertinent university updates. Research steering group meetings are also an opportunity for key areas of concern, progress or questions to be raised by both the WIPS academic lead and Sport Wales Institute leads. As such, these quarterly meetings provide an opportunity for the priorities of the Sport Wales Institute (on behalf of athletes, coaches, practitioners and performance advisors) to be raised and considered.

Beyond attending quarterly RSG meetings, the academic members of WIPS review brief research proposals (generated by the Sport Wales Institute team and/or WIPS research assistants based on conversations with athletes, coaches, practitioners and performance advisors or submitted by external academics) and provide feedback to the proposer. Proposals are reviewed with consideration to pre-existing academic literature available on a topic, potential benefit to performance, anticipated buy-in from potential participants, time requirements and the overall need indicated by the Sport Wales Institute. This review may result in a shift in focus of a proposal (i.e. from a proposed experimental study to a rapid review of literature when an area is already well researched), an enhancement of protocols, a proposal of key academics to engage in the project and/or the academic committing to supporting and/or conducting the proposed research.

In most instances, the proposed research projects (if approved for support by WIPS) are led by the WIPS research assistants with the appropriate academic lead providing oversight, but in some instances the academic may seek funding (i.e. for an MSc or PhD student) or allocate their own research time to conduct the proposed study/studies. Additionally, academic leads respond to requests from members of the Sport Wales Institute as well as coaches and performance directors from associated sports organisations for insights into topical research areas, through written reports, presentations, infographics and more. Academic leads will also share updates from ongoing research projects at their universities that may have performance implications that would benefit the Sport Wales Institute.

## WIPS as iKT

**Fig. 3:**
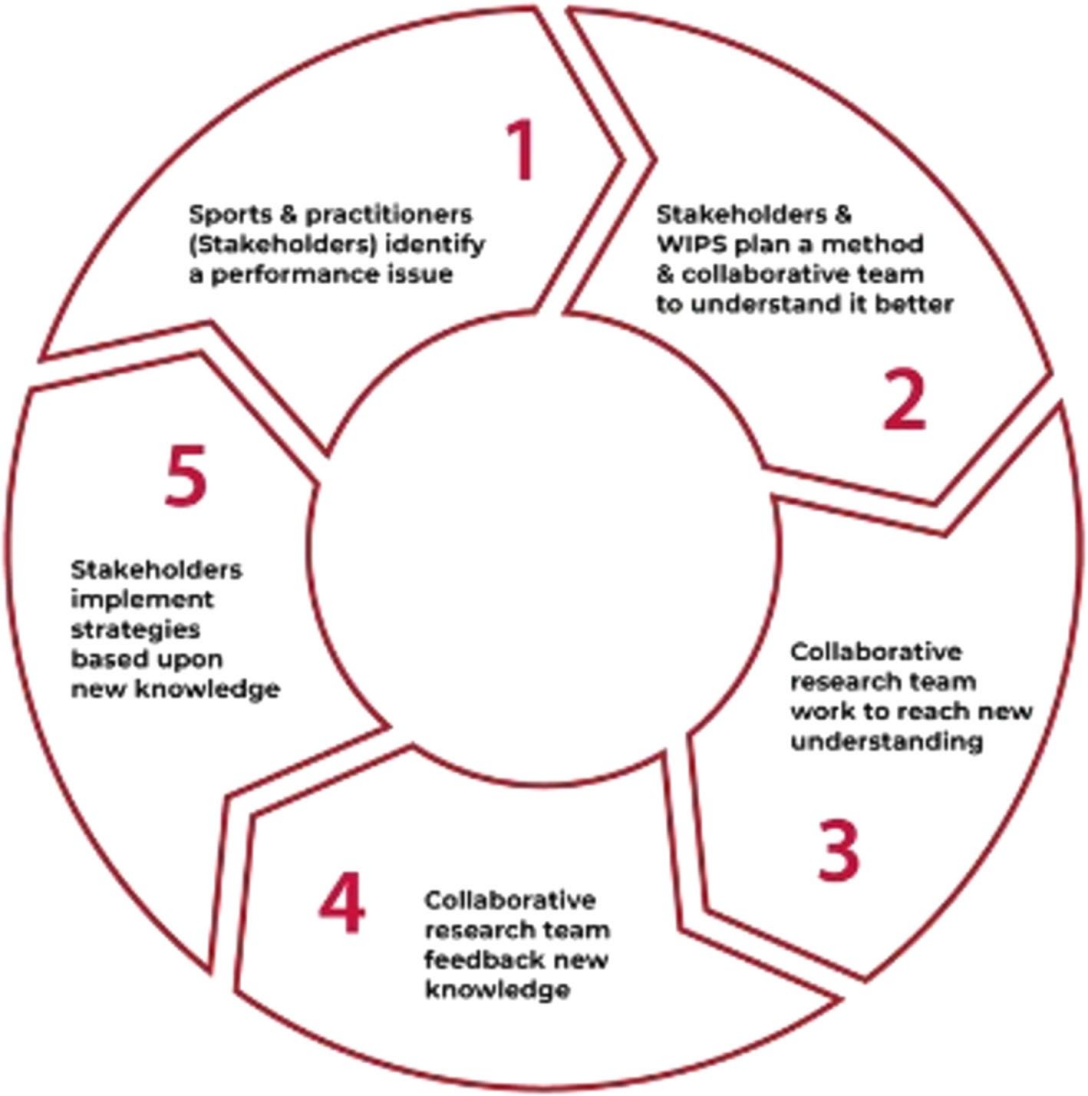
Welsh Institute of Performance Science (WIPS) integrated knowledge translation model*.* Reproduced from [22], with permission

Functionally, iKT at WIPS was conceptualised in a cyclical five-part iterative approach (Fig. 3). First, sport partners (organisations or individual practitioners) identify performance issues through independent reflection, internal discussions with Sport Wales Institute members and/or WIPS research assistants, or WIPS-facilitated workshops. Second, the RSG and relevant sport partners co-design a method and designate key team members to explore the issue. Depending on the scope of the project, this includes aspects such as literature reviews if there was already a body of work completed on a topic, or designing original research if there is little existing or relevant information. Third, the designated research team work to better understand the evidence generated and identify its relevance to the sport partners. Fourth, the team then work to translate the evidence into useful knowledge products or outputs for the organisation based on their own experience and in consultation with other sport partners. Fifth and finally, sport partners implement the knowledge into their practice and the impact is monitored.

The following example illustrates these five steps and the iterative nature of the iKT approach. In 2018, Sport Wales launched its most recent strategy centred on a vision that everyone should be given the opportunity to have “a lifetime of enjoyment through sport”. There was concern from practitioners that elite sport might be seen as separate to this vision. The psychologists and coach developers within the Sport Wales Institute therefore began reviewing the environments being created for athletes across their sports [23]. Specifically, through their work with different sport organisations, they recognised that some organisations were creating environments in which athletes were excelling and seemed to be enjoying their engagement, while others appeared to be more challenging. As such, they recognised that there was a need to review the literature regarding sport environments and subsequently support coaches and performance directors to develop environments in which athletes could thrive. The practitioners wanted to ensure that environments to which athletes were exposed would enable them to thrive, and that Sport Wales’ vision was also upheld in elite sport, ensuring everyone had a lifetime of enjoyment.

The psychology practitioners along with other key individuals submitted an expression of interest to WIPS for support on this project. Two members of the RSG reviewed the expression of interest and established that this was an area that WIPS could support based on it being a priority for the Institute practitioners, being a well-researched area and with a clear performance focus. One of the WIPS research assistants was assigned to the project, along with a lead academic from the RSG. The proposers of the project from Sport Wales subsequently met with the RA and RSG academics on several occasions to explore the scope of the project and the desired outcomes for Sport Wales. Subsequently, the boundaries of the project were established, both in terms of the research to be conducted and the dissemination methods. The RA subsequently engaged in a scoping review of literature pertaining to thriving environments, which was shared with the Sport Wales practitioners (but remained an internal document because of the focus of the review on identifying specific environmental changes that may support the enhanced performance of Welsh athletes).

From here, observations of different sport environments, and informal and formal interviews with athletes, coaches and practitioners were conducted. Regular meetings occurred between the academic team (including the RA) and the Sport Wales Institute team, to keep them updated on the progress of the project. On conclusion of the research, the findings from the scoping review, along with the data obtained by the RA, were integrated and the core characteristics of a “thriving environment” as it applied in sports in Wales were established. Reports of the findings, presentations and infographics were produced to share the insights, with the RA and a psychologist from the Sport Wales Institute leading this. Subsequently, the thriving environments project has been integrated into the overall athlete environment theme of Sport Wales, providing the foundation for policy and practice in this area [24]. It is important to note that these documents are retained internally by Sport Wales, owing to the potential performance benefit associated with them.

## Impact of WIPS

From an applied perspective, WIPS projects have had a broad impact on sport in Wales. Beyond the thriving environments described above, WIPS projects have covered topics including female athlete health, coach and athlete mental health and well-being, injury prevention through psychological and nutritional strategies, identifying kicking cues for elite swim coaches to use in training, developing a field-based measure of trunk strength and parents’ perceptions of athlete development. For overviews of these and other exemplar projects, see https://www.swansea.ac.uk/sport-exercise-sciences/astem/wips/projects/. These projects all resulted in changes to practice or policy for allied sport organisations, demonstrating the range of impact and expertise available through the WIPS. For example, ongoing research and collaboration regarding athlete heat maintenance [18, 25] have had a major impact on UK athletes on the world stage, with coaches crediting Commonwealth Games medals and improved Olympic performances and rankings to the innovations [26]. Likewise, WIPS collaborations led to the Welsh Youth and regular Commonwealth Games teams alike adopting hot baths for heat acclimatisation for the Gold Coast 2018 [27, 28], which has subsequently been recommended in an Expert Statement by the British Academy of Sport and Exercise Sciences [29], and informed programmes for referees in preparation for the 2022 FIFA World Cup in Qatar [30]. It is likely that the broad expertise among the WIPS academic partners facilitated the integration of the WIPS with Sport Wales, as it became a ‘one stop shop’ for sport and performance science knowledge.

WIPS has also made a substantial impact on the academics involved, something which is often missing from accounts of KT as the focus is typically on the impact for the organisation. Between 2015 and 2020, the WIPS team completed 34 research projects (internally dubbed ‘performance projects’) resulting in eight press releases and 28 publications with WIPS-affiliated authors (for a selection, see [18, 25, 31]. Projects affiliated with WIPS received in the region of £200,000 in funding to support studentships and impact dissemination events. Since renewing the WIPS partnership with Sport Wales in 2020, WIPS has completed, or is in the process of completing, 56 further performance projects, resulting, so far, in 56 articles with WIPS-affiliated authors (for a selection, see [32–38]), two press releases [e.g. 39] and approximately £280,000 further in funding through studentships, grants and impact funding. In total, WIPS has engaged in 90 performance projects, supported, or are in the process of supporting, over ten doctoral researchers and ten MSc researchers, obtained over £480,000 in funding, and WIPS-affiliated authors have published 84 peer-reviewed journal articles on WIPS-related projects. Evidently iKT programmes not only help practice but can also result in comprehensive research programmes for academics. Furthermore, as an iKT programme, WIPS is helping to ensure that the value of sport science as an applied discipline is evident.

## Considering Challenges of iKT in WIPS

Sharing WIPS’ approach to iKT presents a unique opportunity to springboard other iKT organisations in sport and performance science and may be broadly generalisable to other settings like physical activity. However, WIPS has not been without its challenges. For example, the second driving principle of WIPS (performance-driven questions, industry-led answers) has failed to materialise to date. Given the role of industry in improving sport outcomes (e.g. innovations in equipment such as improved helmets and headgear or alterations to shoe stiffness for sprinters), there has been surprisingly little interest from industry in partnering with WIPS on research projects. As WIPS does not engage in marketing, it could be that the sport industry is unaware of WIPS’ role in facilitating and supporting research. Furthermore, WIPS is funded through Sport Wales in 4-year cycles, meaning that despite the impact of WIPS on Welsh sport, as the fourth year of each funding cycle approaches, a slight shift in focus occurs and consideration is given as to whether projects that are started will have a chance to finish if funding is not renewed. This leads to a downturn in iKT productivity and an increased focus on communicating (rather than achieving) an impact.

In addition to the limitations of WIPS, there have also been some challenges in the WIPS approach to iKT, often associated with communication. For instance, in some of the earlier projects that were supported by WIPS or projects proposed by academics outside of WIPS, the fourth and fifth steps of the WIPS iKT process (i.e. feeding back and implementing new knowledge) were limited. Specifically, when partnering with external academics, WIPS has encountered issues of academics perceiving that the most important outcome of a project is the publication of an article and one brief report for Sport Wales, rather than engaging in novel and tailored dissemination approaches. Similarly, when knowledge has been disseminated, there are still instances where practitioners, because of a lack of buy-in, time or understanding, have not implemented the findings into their practice.

Last, a particular challenge in proposing WIPS as an example of iKT has been to neatly map the WIPS approach to any one model of KT. For example, when we consider the KtA cycle next to the WIPS approach, differences are also evident. Namely, the knowledge funnel is the first component of the KtA cycle [12], but in the WIPS programme knowledge products are developed following an assessment of project partner needs and are tailored for users during development, not after. This also means the primary knowledge that has been generated has been tailored, and synthesis carried out with a specific focus on solving a performance-driven question. Put another way, if we were to consider the KtA framework alongside what has happened with the WIPS research projects, we would see an iterative interaction between the first three stages of the KtA cycle (problem identification, consideration of barriers to knowledge use and tailoring of knowledge products) and each stage of the knowledge funnel.

While the authors of the KtA cycle do note that movement between the knowledge funnel and action cycle can happen iteratively, [12] our experiences would support that it is perhaps one larger cycle that includes knowledge generation, synthesis and tailoring embedded in the action cycle. Likewise, the applied research model for sport sciences [2] could broadly be applied to the WIPS model, but the applied research model for sport sciences prioritises researcher-driven questions as “athletes and coaches do not always appreciate what they are doing” (p. 256). The WIPS model, however, prioritises the voices of coaches, athletes and sport practitioners, under the assumption that they are more likely than researchers to know what they need, and that by addressing their specific needs, completed projects will have a sustained impact on performance. As such, although the WIPS process has been evidence based, there have been substantial deviations that appear to us to reflect the distinct need of a model of KT specific to sport.

## Conclusions: Future Directions for iKT in Sport

It has been nearly 10 years since WIPS launched and the programme serves as a strong example of iKT in elite sport. With dozens of completed projects benefitting practitioners and sport performers, and nearly as many research outputs, the WIPS model demonstrates that iKT is a mutually beneficial process for researchers and practitioners. Research exploring how WIPS (and other iKT programmes) have aligned with or strayed from planned models of iKT to establish sport-specific KT frameworks would prove valuable for refining our practices. We encourage researchers to explore how they might borrow from or build on the WIPS model to bridge the research-to-practice gap and positively impact elite sport.

## Declarations

### Availability of Data and Material

There are no data associated with this article. However, WIPS annual reports are available from: https://www.swansea.ac.uk/sport-exercise-sciences/astem/wips/.
